# Evaluation of *Trichoderma* spp. on *Fusarium oxysporum* f. sp. *asparagi* and *Fusarium* wilt Control in Asparagus Crop

**DOI:** 10.3390/plants12152846

**Published:** 2023-08-01

**Authors:** Alexandri María Brizuela, Laura Gálvez, Juan Manuel Arroyo, Silvia Sánchez, Daniel Palmero

**Affiliations:** Department of Agricultural Production, Escuela Superior de Ingeniería Agronómica, Alimentaria y de Biosistemas, Universidad Politécnica de Madrid, 28040 Madrid, Spain; alexandri.brizuela@alumnos.upm.es (A.M.B.); laura.galvez@upm.es (L.G.);

**Keywords:** biological control agents, commercial biopesticides, rhizosphere colonization, soilborne disease

## Abstract

Among the key diseases affecting the asparagus crop (*Asparagus officinalis* L.), vascular wilting of asparagus caused by *Fusarium oxysporum* f. sp. *asparagi* stands out worldwide. This disease significantly shortens the longevity of the crop and limits economic production. Traditional control measures have been largely ineffective, and chemical control methods are difficult to apply, making biological control approaches, specifically the use of *Trichoderma*, an economical, effective, and risk-free alternative. This study aimed to identify the main factors that affect the efficacy of biopesticides studied as Biological Control Agents (BCAs) against Fusarium wilt in asparagus and to assess the efficacy of *Trichoderma*-based biopesticides under greenhouse and semi-field conditions. We evaluated the response of three *Trichoderma* spp. (*T. atroviride*, *T. asperellum*, and *T. saturnisporum*) to environmental variables, such as temperature and water activity, and their antagonistic capacity against *Fusarium oxysporum* f. sp. *asparagi*. All three *Trichoderma* species inhibited the growth of the pathogen in vitro. A decrease in water activity led to a greater reduction in the growth rate. The efficacy of the three biological control agents decreased with higher temperatures, resulting in minimal inhibition, particularly under conditions of restricted available water in the environment. The effect of the fungal inoculum density was also analyzed at two different temperatures. A direct correlation between the amount of inoculum and the score on the Disease Severity Index (DSI) was observed. A notable reduction in DSI was evident in treatments with high inoculum density (10^6^ conidium/mL) for all three species of *Trichoderma* tested at both temperatures. In greenhouse and semi-field tests, we observed less disease control than expected, although *T. asperellum* and *T. atroviride* showed lower disease severity indices and increased the dry weight of seedlings and crowns, whereas *T. saturnisporum* resulted in the highest disease rate and lowest dry weight. This work highlights that the efficacy of *Trichoderma* as BCAs is influenced by various factors, including the quantity of soil inocula, and environmental conditions. The study findings have strong implications for selecting appropriate *Trichoderma* species for controlling specific pathogens under specific environmental conditions.

## 1. Introduction

Asparagus (*Asparagus officinalis* L.) is a leading outdoor horticultural crop worldwide, with a gross production value of USD 12,655 annually [1]. In Europe, it is grown on over 62,000 ha, of which 60% is present in two countries: Germany and Spain. Spain is the second-largest producer of asparagus in Europe, producing 65,000 tons across 14,020 ha [2].

Fusarium Vascular Wilt, caused by *Fusarium oxysporum* f. sp. *asparagi*, is one of the primary diseases that affect asparagus crops. Diseased plants exhibit a gradual loss of vigor [3,4,5], which is accompanied by a considerable decrease in yield. In the advanced stages of the disease, plants exhibit premature yellowing, ultimately resulting in their death by the end of the cycle [4,6].

Cultivation practices considerably complicate disease control [4,5,7]. Throughout the harvest months, all radiating shoots below ground level were cut using a knife. Wounds sustained during harvesting provide a direct path for soil-borne pathogens to reach the plant’s vascular system, thereby promoting fungal colonization [6,8].

Controlling this disease is challenging owing to the perennial nature of asparagus crops and the fungus’s ability to survive for extended periods in both plant debris and soil. The standard control measures used for horticultural crops are often ineffective or challenging to apply to asparagus crops [9]. To date, no resistant plant material has been identified for Fusarium Vascular Wilt in asparagus crops [10,11,12,13,14,15]. Chemical control methods have also proven to be challenging to apply during the harvest period. They are also ineffective when the soil has a high inoculum density [16].

Biological control approaches using several yeasts, fungi, and bacteria are effective, economical, and risk-free alternatives to the use of chemical pesticides against plant diseases; among them, *Trichoderma*, a mycoparasitic fungus, is noteworthy [17,18]. Bioantagonists protect plants from phytopathogens by inhibiting or suppressing disease development before or after infection [19].

Weindling [20] first identified *Trichoderma* as a biological control agent. While this genus effectively inhibits the growth of many plant pathogenic fungi, such as *Rhizoctonia*, *Fusarium*, *Pythium*, *Sclerotium*, and *Phoma*, this capacity varies genetically between species [17,21,22,23,24].

*Trichoderma* spp. colonize and grow on developing roots that promote normal plant development [25]. They also compete with pathogenic fungi for space and nutrients by colonizing the rhizosphere, thereby directly suppressing disease progression [26,27]. Additionally, it also induces systemic antioxidant enzyme activities, upregulating the expression of class III plant peroxidase and catalase, as well as phenolics and proteins, such as salicylic acid pathway-related genes and pathogenesis-related protein 5, or jasmonic acid-related gene 12-oxo-phytodienoic acid reductase, which are associated with resistance to *Fusarium* [28,29,30].

*Trichoderma* has a direct effect on pathogenic fungi through the production of antibiotics and lytic enzymes such as cellulases, hemicellulases, xylases, and chitinases [31]. Many bacteria directly act against enzymes produced by pathogens during the infection process. For example, *T. harzianum* secretes Trichodermamine B, while *T. virens* produces viridin, virone, and trichosetin, which act as potential inhibitors of the cell wall-degrading enzyme polygalacturonase secreted by *F. oxysporum* f. sp. *lycopersici* [26,32,33,34,35,36,37,38,39,40,41,42]. *Trichoderma* has also been reported to have the ability to degrade mycotoxins produced by *Fusarium* through the upregulation of the UDP-glucuronosyltransferase gene.

Various *Trichoderma* species, including *T. atroviride*, *T. asperellum*, *T. gamsii*, *T. harzianum*, *T. afroharzianum*, and *T. atrobrunneum*, have been registered as commercial biopesticides. Many of these species have been studied in various pathosystems [43,44,45,46]. However, different species respond differently to different pathogens and environmental conditions. To achieve effective disease control, the choice of a biological control agent should consider both the pathogen’s characteristics and the relevant climatic factors. Selecting appropriate *Trichoderma* spp. as BCA is crucial in determining their response to different environmental conditions. Although there are many studies on *Trichoderma* spp., some have focused on soil fungi, and there are no data on their efficacy against *Fusarium oxysporum* f. sp. *asparagi.* Furthermore, disease control effectiveness was not affected by either the amount of inoculum from the pathogen or the dose of the commercial product.

This study aimed to identify the main factors that affect the efficacy of biopesticides studied as BCAs against *Fusarium* wilt in asparagus. In vitro and plant bioassays were conducted to (i) assess the antagonistic capacity of three *Trichoderma* species against the mycelial growth of *Fusarium oxysporum* f. sp. *asparagi* and how this is affected by temperature, water activity, and their interactions. (ii) analyze the impact of the amount of inoculum on the efficacy of the three *Trichoderma* species on seedlings, and (iii) evaluate the effectiveness of two commercial formulations registered in the EU under greenhouse and semi-field conditions.

## 2. Results

### 2.1. Effect of Different Environmental Variables on the Antagonistic Capacity In Vitro

**Figure 1:**
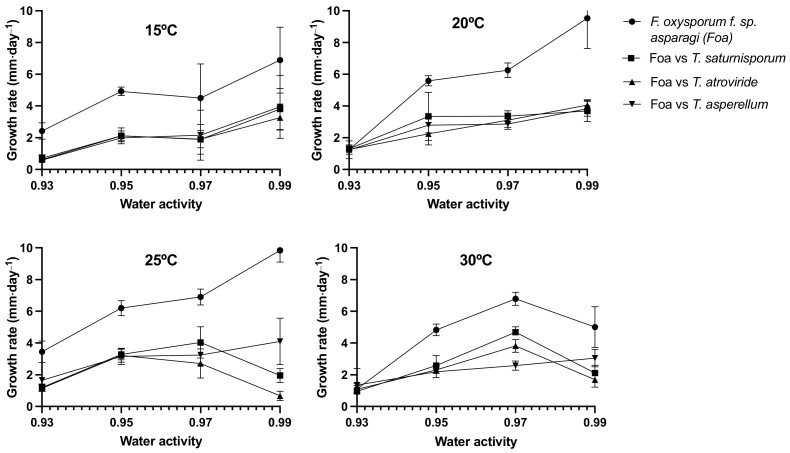
Daily fungal growth rate for *Fusarium oxysporum* f. sp. *aspargi* was calculated for each fungal matrix and control.

The results showed an inhibitory effect on the mycelial growth of *F. oxysporum* f. sp. *asparagi* against the three *Trichoderma* spp. analyzed. Different climatic variables considerably affected the growth rate of the pathogen (Figure 1). Generally, a decrease in water activity led to a greater reduction in the growth rate. The pathogen’s growth rate was highest under less restrictive environmental conditions (20–25 °C with high water activity), but it was also more strongly inhibited under these conditions in dual cultures with *Trichoderma* species. At 25 °C and a_w_ = 0.99, dual cultures with *T. atroviride* showed the greatest reduction in growth rate, up to 93.2%. The efficacy of the three biological control agents decreased with higher temperatures, resulting in minimal inhibition, particularly under conditions of restricted available water in the environment (Figure 1).

We used the General Lineal Model method to estimate the repeatability and reproducibility of mycelial growth of *Fusarium oxysporum* f. sp. *asparagi* and identified five effects: temperature, days, water activity, and second- and third-order interactions. The model allowed for mycelial growth of FOAS alone (R-Squared = 90.11%, adjusted R-Squared = 90.06%) or in dual culture with different BCAs: *T. saturnisporum* (R-Squared = 67.57%, adjusted R-Squared = 67.19%), *T. asperellum* (R-Squared = 82.92%, adjusted R-Squared = 82.73%), and *T. atroviride* (R-Squared = 79.53%, adjusted R-Squared = 79.31%). All species exhibited similar quality, where growth was evidently inhibited when the pathogenic isolate was subjected to any of the three *Trichoderma* species, showing higher inhibition rates under higher aw conditions and decreased inhibition with decreasing water activity.

**Figure 2:**
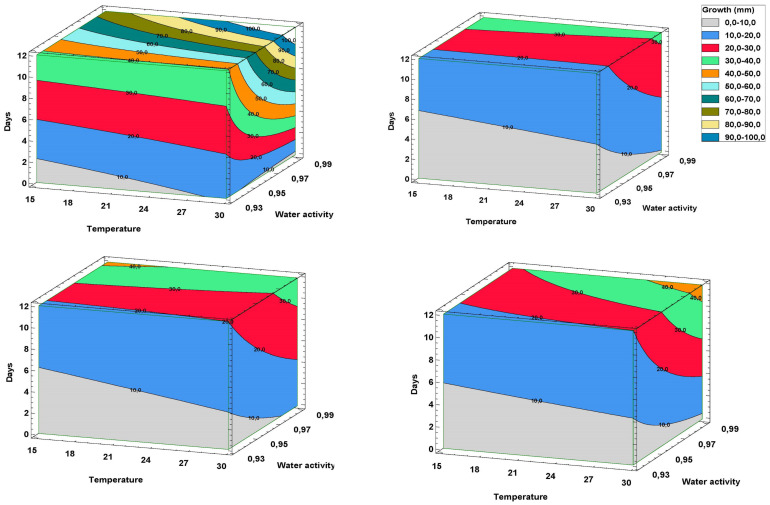
Surface contour plot of daily growth rate responses at varying temperatures and water activities. *Fusarium oxysporum* f. sp. *asparagi* (Foa): (**up**–**left**); Foa vs. *T. saturnisporum*: (**up**–**right**); Foa vs. *T. asperellum*: (**down**–**left**); Foa vs. *T. atroviride*: (**down**–**right**).

Modeling a disease caused by a soil fungus is challenging, but contour plots are useful for determining response values. The 3D surface contour plot depicted in Figure 2 aided in comprehending the relationship between the key factors and mycelial growth response values. The gray region showed the lowest mycelial growth, which decreased with increasing temperature and water activity. *T. atroviride* displayed orange regions (indicating growth of over 40 mm) under high temperature and water activity, while subtle differences were observed between *Trichoderma* spp. under high water activity. However, such rates are only observed at temperatures below 20 °C in dual cultures with *T. asperellum*. In contrast, the model estimated a much lower growth rate at 12 d in dual cultures of *T. saturnisporium*.

At the highest water activity (a_w_ = 0.99), dual cultures were categorized into classes 1 and 2 using Bell’s scale, indicating the greatest inhibition rates. However, at the lowest water activity (a_w_ = 0.93), all plates were classified between classes 3 and 4 (Figure 2). *T. saturnisporum* and *T. atroviride* exhibited lower levels on the Bell scale for the temperature and water activity combinations analyzed, except at the lowest water activity. *T. asperellum* was less effective, with Bell’s index values exceeding two in three out of four tested water activities (Table 1).

### 2.2. Effect of the Fungal Inoculum Density on the Efficacy of Trichoderma *spp.*

**Figure 3:**
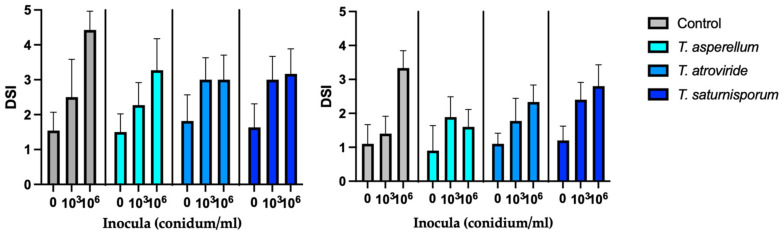
Disease severity index of seedlings pretreated with *Trichoderma* spp. and different inocula density depending on the temperature: 25 °C (**Left**) and 30 °C (**Right**).

We analyzed the impact of inoculum quantity in the substrate on disease symptom manifestation during early development stages at two different temperatures. We observed a direct correlation between the amount of inoculum and DSI, with high exponential correlations at both temperatures (y = 0.569·e^0.5543x^, R-Squared = 0.941 at 25 °C; y = 0.828·e^0.5413x^, R-Squared = 0.980 at 30 °C). Application of *Trichoderma* did not result in any statistically significant differences (*p* < 0.001) compared to the untreated control or lower inoculum density. Nonetheless, a notable reduction in the severity index was evident in treatments with high inoculum density (10^6^ conidium·mL^−1^) for all three species of *Trichoderma* tested at both temperatures (Figure 3).

In the experiments conducted at 25 °C, applying three *Trichoderma* spp. resulted in a decrease in DSI. *T. asperellum* showed the lowest DSI, achieving over a 50% reduction compared to the inoculated control, followed by *T. atroviride* and *T. saturnisporum*. At higher temperatures, reductions in severity index varied from 26.09 to 32.2% across all three *Trichoderma* species.

### 2.3. Effectiveness of Trichoderma *spp.* on Commercial Products under Greenhouse and Semi-Field Conditions

**Figure 4:**
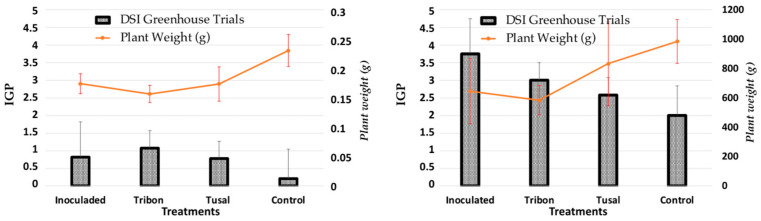
Dry weight and Disease Severity Index (DSI) in pretreated asparagus plants after inoculation with FOA in Greenhouse trials (**Left**) and Semi-field trials (**Right**).

We analyzed two commercial products for controlling soil-borne diseases available in Europe. Early-stage plants were evaluated in greenhouse trials, and fully developed plants were evaluated under semi-field conditions. Neither of the analyzed products showed a decrease in DSI in 8-week-old inoculated seedlings (Figure 4). No significant differences were observed between the dry weights of the seedlings treated with the two commercial products and those of the inoculated control plants.

However, considerable differences were observed when the trial was extended to semi-field conditions (in large outdoor pots) for 18 months compared to the inoculated plants.

Plants pretreated with Tusal^®^ (*T. asperellum* and *T. atroviride*) at a dose of 1 kg/ha showed lower disease severity indices than the inoculated controls to some extent. In contrast, plants pretreated with Tribon^®^ (*T. saturnisporum*) showed fewer symptoms of the disease to a lesser extent, without significant differences from the inoculated controls, although with lower mean DSI values.

**Figure 5:**
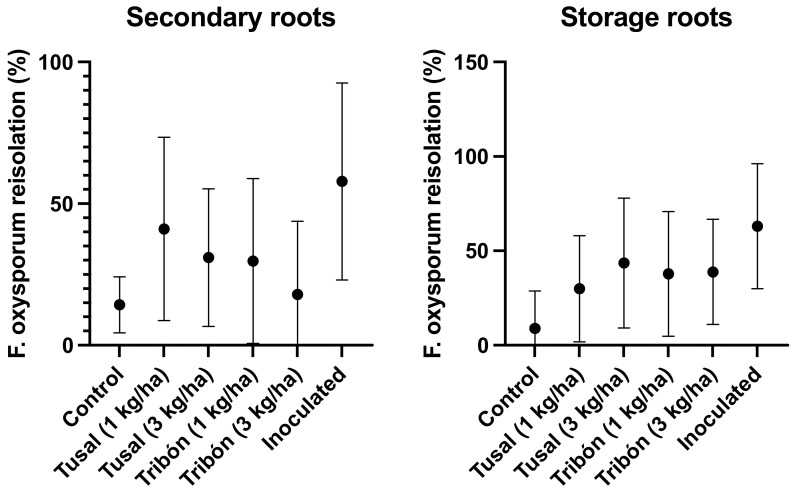
Re-isolation percentages of *F. oxysporum* f. sp. *asparagi* and Disease Severity Index (DSI) depending on the dose of the *Trichoderma*-based commercial product.

Irrespective of dosage, the test results consistently showed that the pathogen affected a higher percentage of secondary roots compared to reserve roots. However, using a higher dose (3 kg·ha^−1^) decreased this effect, though the disease was never fully suppressed and the recorded DSI remained above two. However, inoculation treatment consistently resulted in a higher percentage of isolation for both types of rot (Figure 5).

## 3. Discussion

*Trichoderma* is widely used as a biological control agent to mitigate pathogenic activity and promote plant growth [47,48]. In many cases, it has proven to be an effective alternative to fungicides [49]. However, its efficacy for many crops is under discussion [50,51,52], which can be influenced by various factors, such as soil type, host crops, and climate.

Several studies evaluating the antagonistic activity of *Trichoderma* strains have reported high inhibition rates against pathogenic fungi in vitro. However, when tested in vivo, the results did not consistently demonstrate the same antagonistic effects [19].

We obtained promising results for mycelial growth inhibition in an in vitro study in which different *Trichoderma* spp. inhibited the growth of the pathogen. In vitro inhibitory effects have been demonstrated for the three *Trichoderma* species studied, although results show how environmental factors such as water activity or temperature modify such efficacy. These results indicate that the selection of the appropriate *Trichoderma* must be made based on specific characteristics, with temperature acting as a key factor.

Greenhouse (seedlings and plants) and semi-field tests showed less disease control than expected. In a study on asparagus seedlings, *Trichoderma harzianum* could not reduce the severity of the vascular disease [16]. González et al. [53] found no considerable differences between asparagus plants treated with *Trichoderma* spp. and the untreated control in terms of the effects of *Fusarium oxysporum* f. sp. *asparagi* and *F. solani.* However, these authors reported that plants treated with *T. atroviride* and *T. asperellum* in interaction with pathogenic fungi showed better results in the weight of the asparagus plants compared to the uninoculated *Trichoderma* spp. treatments. 

Arriola et al. [54] successfully suppressed the disease and increased dry weight by combining *T. harzianum* with arbuscular mycorrhizae. In contrast, Rubio-Pérez et al. [55] observed an increase in root and aerial development and a positive antagonistic effect on *F. oxysporum* and *F. proliferatum* by using a combination of *T. asperellum* and *T. harzianum* previously isolated from the asparagus rhizosphere.

Our study showed that all three *Trichoderma* BCAs reduced DSI, with *T. asperellum* at 25 °C achieving the highest reduction (50%). At higher temperatures, the DSI reduction ranged from 26.09 to 32.2% for all three antagonistic *Trichoderma* species. Additionally, application of *T. asperellum* and *T. atroviride* (Tusal^®^) increased the dry weight of seedlings and crowns, as seen in Figure 5.

*Trichoderma asperellum* is effective against other soil-borne pathogens, such as *Fusarium oxysporum* in tomatoes, while promoting plant growth [38]. Similarly, Rao et al. [56] considered the use of *T. atroviride* (strain LZ42) as an effective alternative for the integrated management of wilt disease caused by *Fusarium* spp. In the case of *T. saturnisporum*, few studies have been conducted on its use in pepper plants. However, Diánez Martínez et al. [57] found that applying this microorganism stimulated seedling growth and suppressed the severity of soil-pathogenic fungi such as *Phytophthora capsici* and *Phytophthora parasitica*. Pretreatment efficacy appears to be highly influenced by the virulence of the pathogen and the quantity of soil inocula. Our results show that at the highest inoculum density of the pathogenic fungus tested (10^6^ conidium·mL^−1^), a substantial decrease in the DSI was observed for the three *Trichoderma* spp. at the two temperatures studied.

## 4. Materials and Methods

### 4.1. Effect of Climatic Variables on the In Vitro Antagonism of Trichoderma *spp.* and Fusarium oxysporum *f. sp.* asparagi (FOA)

The response of three *Trichoderma* spp. (*T. atroviride*, *T. asperellum*, and *T. saturnisporum*) to environmental variables, such as temperature and water activity, and their antagonistic capacity against *Fusarium oxysporum* f. sp. *asparagi* (isolate FOA01) were evaluated by using dual in vitro cultures [58]. All isolates used were collected from the Plant Protection Laboratory (ETSIAAB-UPM) [4]. A completely randomized factorial experimental design (3 × 4 × 4 × 6) was employed, where the first factor was the BCAs and the second and third factors were temperature (15, 20, 25, and 30 °C) and water activity (a_w_), respectively. 

To modify the a_w_ value of potato dextrose agar (PDA) (Merck, Darmstadt, Germany), we added glycerol as a nonionic solute to obtain a_w_ values of 0.97, 0.95, and 0.93. Glycerol was not added to the control group (a_w_ = 0.996). The a_w_ of the medium was measured using a hygrometer (AquaLab 3TE; Decagon Devices Inc., Pullman, WA, USA).

Six replicates of 90 mm Petri dishes with dual-cultures (Isolate FOA01—*Trichoderma* spp.) were prepared for each combination of isolate, temperature, and water activity. After incubation in a growth chamber (EQUITEC Brand; ERI 40405 3S LED model, Spain), with a temperature and photoperiod regulated at 25/18 °C day/night, the radial growth of the mycelium (mm) was measured every 24 h using a digital vernier. Growth inhibition percentage was calculated based on the formula proposed by Royse and Ries [58] as follows: PICR% = (R1 − R2)/R1 × 100, where PICR is the percentage inhibition of radial growth and R1 is the radial growth (mm) of the pathogen without *Trichoderma* spp. R2 = radial growth (mm) of pathogens with *Trichoderma* spp. The daily fungal growth rate was calculated for each fungal matrix and expressed as mm·d^−1^ [59,60,61].

Dual cultures were rated on a scale of 1 to 5 according to the Bell scale based on the plate surface colonized by *Trichoderma* and the pathogen [62].

### 4.2. Antagonistic Effect of BCAs on Asparagus Seedlings Inoculated with Fusarium oxysporum *f. sp.* asparagi (FOA)

Three-week-old seedlings of cv. Grande sown in alveoli (138 cc) containing sterile peat were pretreated with 5 mL of *Trichoderma* suspension (containing 10^10^ conidia·mL^−1^). Four weeks after sowing, six pretreated seedlings per treatment were inoculated with 5 mL of the pathogenic fungus (isolate FOA01) and incubated in a growth chamber (EQUITEC Brand; ERI 40405 3S LED model). Plants were fertilized for 15 days after sowing using a water-soluble NPK 7-5-6 universal fertilizer (COMPO^®^) at a dose of 5 mL·L^−1^. Plants were slightly watered every other day. The experiment included non-inoculated controls and was repeated twice for each inoculum concentration tested (0, 10^3^, and 10^6^ conidia·mL^−1^) and temperature (25 and 30 °C). Ten weeks after sowing, the weight and disease severity indices were measured. The disease severity index (DSI) in asparagus plants was evaluated according to the scale proposed by Stankovic et al. [63], where 0 = plants without disease symptoms, 1 ≤ 10% root rot, 2 = 10–50% root rot, and 3 ≥ 50% root rot, 4 = roots completely rotten. At the end of each experiment, the samples were dried in an oven (Digiheat 80 L; Model 2001244, Fleurus, Belgium) at 60 °C for 48 h, and the dry weights of the roots and aerial parts were recorded separately.

### 4.3. Efficacy of Commercial Biopesticides

#### 4.3.1. Greenhouse Trials

Three-week-old seedlings of cv. Grande were transplanted into 530 cc pots and treated with 20 mL of commercial biopesticides based on different species of *Trichoderma*. The biopesticides used were Tusal^®^ (consisting of *T. asperellum* (T25)—0.5% (10^8^ CFU·g^−1^) and *T. atroviride* (T11)—0.5% (10^8^ CFU·g^−1^), Tribon^®^ (*T. saturnisporum* strain TSMIP01 (10^12^ CFU·l^−1^), and Potassium 3.5% *w*/*w*), applied in doses of 4 g·dm^−3^ and 4 mL·dm^−3^, respectively. The experimental unit contained two repetitions with five plants each. The test was repeated. Five weeks after sowing, pots were inoculated with a 20 mL conidial suspension of three isolates of *Fusarium oxysporum* f. sp. *asparagi* adjusted to 10^6^ conidia·mL^−1^, coded as FOA01, FOA11, and FOA28. The DSI and dry weight of the plants were analyzed.

#### 4.3.2. Semi-Field Trials

The same commercial products (Tusal^®^ and Tribon^®^) were tested in a semi-field trial. Asparagus crowns (80–100 g) of cv. Grande were planted in outdoor fiber cement pots (0.1 m^3^) at two doses: 1 kg·ha^−1^ (equivalent to 0.05 mg·m^−2^) and 3 kg·ha^−1^ (equivalent to 0.015 mg·m^−2^). Two weeks after sowing, *Fusarium oxysporum* f. sp. *asparagi* (FOA01 isolate) was inoculated by irrigation, adding 100 mL of the inoculum per pot at a concentration of 5 × 10^6^ conidia·mL^−1^. Non-inoculated controls were included in the experiment, and booster treatments were added with commercial biopesticides at the same dose every three months. Aerial biomass dry weight and rot severity index were evaluated in crowns and roots after 18 months. Analysis of isolates of *F. oxysporum* f. sp. *asparagi* from treated plants involved superficial disinfection of secondary and stored roots with a 1.5% sodium hypochlorite solution for 1 min, followed by two successive washes with sterile distilled water. After drying, 1 cm pieces were sown in plates with potato dextrose agar (PDA) culture medium supplemented with 0.5 g·L^−1^ streptomycin sulfate (Sigma-Aldrich, St. Louis, MO, USA) and incubated for 5–7 days at laboratory temperature (25 °C) under continuous fluorescent light. The procedures and taxonomic criteria described by Nelson et al. [64], and Leslie and Summerell [65] were followed.

### 4.4. Statistical Analysis of Data

Analysis of variance was performed using Fisher’s least significant difference (LSD) test with STATGRAPHICS Centurion XVIII statistical package software (StatPoint, Inc., Herndon, VA, USA, https://www.statgraphics.com/centurion-xviii, accessed on 1 May 2023). Graphics were analyzed using GraphPad Prism (version 9.0 for Windows, GraphPad Software, San Diego, CA, USA, www.graphpad.com, accessed on 1 May 2023). To estimate the correlation between the growth rate at any of the studied temperatures and the water activity, we adjusted a simple regression analysis to the non-linear inverse-X model, as it showed the highest R^2^ value. The same statistical package software was used to construct a generalized linear model of the growth rate (Yi = β0 + β1 × 1, i + β2 × 2, i + β3 × 3, i + … + βk × k, i + εi, where “Y” is the response variable (growth rate), “β1Xk,i” the predictor variables (temperature and water availability), and “i” the error). The linear regression of the increase in radius against time (d) was used to obtain growth rates (mm·d^−1^). The Pearson correlation coefficient at the 5% confidence level was used as the significance criterion.

## 5. Conclusions

The in vitro inhibitory effect on the mycelial growth of *F. oxysporum* f. sp. *asparagi* against the three *Trichoderma* spp. has been proven. The efficacy of *Trichoderma* as BCAs is influenced by various factors, including the quantity of soil inocula and environmental conditions, which will have strong implications for selecting appropriate *Trichoderma* species for controlling specific pathogens under specific environmental conditions.

However, when early-stage and fully developed plants were evaluated in greenhouse and semi-field trials, the inhibitory effects of commercial products were not produced to the same extent. Although, in some cases, positive effects on the crop have been observed and a notable reduction in DSI has been evident, the disease was never fully suppressed.

Full control was not achieved at the tested doses; however, root rot caused by *F. oxysporum* was alleviated to some extent. The efficacy of these treatments has been widely studied and debated. However, the efficacy depends on the host-pathogen-BCA interaction. Finally, to optimize the use of *Trichoderma* as a biocontrol agent, an appropriate integrated pest-management approach is required. This includes tolerant varieties, the use of healthy plant material, and soil disinfection before transplanting to reduce soil inocula. This study will be the basis for a multi-year study carried out in an asparagus growing area under real field conditions, where the scope and limitations of disease control will be determined.

## Figures and Tables

**Table 1:** Bell antagonism scale on the dual cultures with *Fusarium oxysporum* f. sp. *asparagi* and the different *Trichoderma* spp., depending on the temperature and water activity, after 7 days.

Biocontrol Agents	Temperature	Water Activity			
		0.99	0.97	0.95	0.93
*T. saturnisporum*	15	1	1.5	2.6	3
	20	1.6	1	1.1	2
	25	1	1.8	2	3
*p*-Value = 0.00	30	1	1	1	1.8
*T. atroviride*	15	1	1	2	3.8
	20	1	1	1	3.5
	25	1	1	2	4
*p*-Value = 0.00	30	3	3.6	2	4
*T. asperellum*	15	1.6	2.8	3.6	4
	20	2	3	3	4
	25	1	2.6	3	4
*p*-Value = 0.01	30	1	1	3	4

## Data Availability

The data presented in this study are available on request from the corresponding author.
